# Gene therapy for genodermatoses at the crossroads of innovation and clinical translation

**DOI:** 10.3389/fbioe.2026.1901459

**Published:** 2026-08-24

**Authors:** Alessandra Fabrizi, Marta Valenti, Silvia Zacchino, Katia Russo, Erica Baschetti, Laura De Rosa, Michele De Luca

**Affiliations:** Centre for Regenerative Medicine “Stefano Ferrari” Department of Life Sciences, University of Modena and Reggio Emilia, Modena, Italy

**Keywords:** epidermolysis bullosa (EB), gene editing, gene therapy, genodermatoses, ichthyosis, regenerative medicine, stem cells

## Abstract

Inherited genodermatoses are a heterogeneous group of rare monogenic disorders. Among these, epidermolysis bullosa (EB) and ichthyoses represent paradigmatic disorders characterized by severe skin fragility and hyperkeratosis, respectively, and impaired barrier function, often with profound effects on quality of life and systemic health. Current management remains largely palliative, underscoring the urgent need for disease-modifying therapies. Over the past 2 decades, advances in epithelial stem cell biology, vector engineering and genome editing technologies have transformed the therapeutic landscape for genodermatoses. *Ex vivo* gene therapy has provided the first proof that genetically corrected epidermal stem cells can achieve long-term tissue regeneration in EB skin patients, establishing a new paradigm for regenerative medicine. In parallel, the emergence of programmable genome engineering platforms, including CRISPR/Cas nucleases, base editors and prime editors, have enabled increasingly precise strategies for mutation-specific correction in both recessive and dominant disorders. Furthermore, the development of *in vivo* topical approaches is expanding the possibility of directly targeting the skin. Despite these advances, substantial translational barriers continue to limit broad clinical implementation. Efficient and durable targeting of epidermal stem cells within a highly regenerative tissue, together with safe delivery across the skin barrier, stringent control of off-target activity, scalable manufacturing and demonstration of long-term safety, remain major challenges for the clinical translation of these approaches. In this Review, we discuss the current state of gene therapy for genodermatoses, highlighting key clinical milestones, emerging genome editing technologies and next-generation delivery systems. We further examine the biological and regulatory challenges that need to be overcome to bridge the gap between experimental innovation and clinically accessible therapies for patients with inherited skin diseases.

## Introduction

Inherited genodermatoses represent a heterogeneous group of rare monogenic disorders caused by mutations in genes encoding structural or functional proteins, which are essential for the skin homeostasis, integrity and barrier function (Koller and Bauer, 2021). Genodermatoses are associated with a substantial clinical burden due to their severity, chronic progression and lack of curative therapies (Has et al., 2020). Nevertheless, many of these disorders arise from well-defined monogenic defects, making them attractive candidates for gene- and cell-based therapeutic strategies (Morren et al., 2021). In this context, the skin is particularly amenable for these approaches because of its accessibility, which enables both *ex vivo* manipulation of patient-derived cells and *in vivo* topical delivery (De Rosa et al., 2014; Hirsch et al., 2017). To date, the major therapeutic advances have primarily focused on epidermolysis bullosa (EB) and ichthyoses, therefore here we will primarily focus on the therapeutic strategies developed for these two genodermatoses (Koutsoukos and Bilousova, 2024). However, additional inherited keratinization disorders, including palmoplantar keratoderma (PPK), particularly epidermolytic palmoplantar keratoderma (EPPK), peeling skin syndromes, and acantholytic disorders such as Hailey-Hailey disease and Darier disease, are also being investigated as potential targets for emerging gene editing strategies (Brooks et al., 2023).

## Epidermolysis bullosa

EB comprises a heterogeneous group of rare inherited skin disorders caused by more than 1,000 mutations in at least 16 structural genes encoding for proteins forming hemidesmosomes and anchoring fibrils, which are essential for dermal-epidermal adhesion and tissue integrity (Has et al., 2020). Despite its rarity, with an estimated prevalence of approximately 11 cases per million live births, EB represents a spectrum of disorders with highly variable clinical severity (Fine et al., 2008; Fine, 2016). EB is characterized by recurrent blistering with chronic and often non-healing wounds, and secondary infections (Has et al., 2020; Fine et al., 2014). Moreover, extracutaneous manifestations may occur affecting multiple organs, such as the gastrointestinal tract, eyes and musculoskeletal system, contributing to overall disease burden (Has et al., 2020). One of the most severe long-term complications is the development of cutaneous squamous cell carcinoma (SCC), particularly in recessive dystrophic EB and severe form of junctional EB, where it represents the leading cause of mortality (Condorelli et al., 2019; Robertson et al., 2021). Currently, therapeutic options for EB remain largely palliative, focusing on wound care, pain control and prevention of complications (Prodinger et al., 2019). Effective curative therapies are still lacking, highlighting a critical unmet clinical need and driving the development of increasingly targeted gene- and cell-based therapies (Prodinger et al., 2019).

Based on the plane of ultrastructural fragility, EB is classified into four major types: EB simplex (EBS), junctional EB (JEB), Kindler EB (KEB) and dystrophic EB (DEB) (Bardhan et al., 2020). Inheritance varies among these types, influencing the choice of gene- and cell-based therapeutic strategies (Has et al., 2020; Welponer et al., 2021).

### Epidermolysis bullosa simplex

EBS is predominantly characterized by dominant mutations affecting *KRT5* and *KRT14* genes, encoding keratins 5 and 14, which constitute the intermediate filament network of basal keratinocytes (Prodinger et al., 2019; Bardhan et al., 2020). Thus, EBS is characterized by cytoskeletal fragility of basal keratinocytes, leading to intraepidermal blisters (Bardhan et al., 2020). Clinically, the severity of blistering ranges from limited to hands and feet to widespread involvement; additional features can include hyperkeratosis of the palms and soles (keratoderma), nail dystrophy, milia and hyper- and/or hypopigmentation (So et al., 1993).

### Junctional epidermolysis bullosa

JEB is one of the most devastating forms of EB. It is characterised by recessively inherited mutations in genes encoding the heterotrimeric protein laminin 332 (*LAMA3*, *LAMB3*, *LAMC2*), collagen XVII (*COL17A1*), integrins α6β4 (*ITGA6*, *ITGB4*), and integrin α3 (*ITGA3*) (Has et al., 2020). These alterations impair the stability of the dermal-epidermal junction, leading to the formation of subepidermal blisters at the level of the lamina lucida (Bardhan et al., 2020). Clinically, JEB is characterized by blistering with minimal or no trauma, typically presenting at birth or in early infancy (Pfendner et al., 1993). The disease spectrum ranges from severe, often early lethal generalized forms (which may be associated with congenital malformations of the urinary tract and bladder) to milder intermediate phenotypes (characterized by localized blistering of the hands, feet, knees, and elbows, which may include renal or ureteral involvement) (Pfendner et al., 1993).

### Kindler epidermolysis bullosa

Kindler syndrome is caused by recessively inherited mutations in *FERMT1*, the gene encoding fermitin family homolog 1 (kindlin-1), an intracellular protein associated with focal adhesions (Bardhan et al., 2020; Lai-Cheong and McGrath, 2010). Unlike other forms of EB, Kindler syndrome is characterized by impaired actin cytoskeleton-extracellular matrix interactions and a variable plane of blister formation at or close to the dermal-epidermal junction (Lai-Cheong and McGrath, 2010).

### Dystrophic epidermolysis bullosa

DEB is caused by pathogenic variants in *COL7A1*, which encodes type VII collagen, the main structural component of anchoring fibrils (Sakai et al., 1986; Varki et al., 2007). Blisters occur in the dermis, just below the lamina densa of the basement membrane (Bardhan et al., 2020). DEB can be dominantly (DDEB) or recessively (RDEB) inherited (Lucky et al., 1993). RDEB is the most severe form, characterized by generalized skin fragility with trauma-induced blistering from birth, leading to extensive scarring, mucosal involvement, pseudosyndactyly and a high risk of aggressive cutaneous squamous cell carcinoma. DDEB is generally the mildest form, blisters appear mainly on the hands, feet, knees and elbows (Lucky et al., 1993).

## Ichthyosis

Among genodermatoses, ichthyoses share genetic complexity and clinical challenges comparable to the ones of EB. This disorder can be either genetic or acquired, with both forms characterized by a disruption of the skin homeostasis during the formation of the stratum corneum (DiGiovanna and Robinson-Bostom, 2003).

Clinically, ichthyoses are characterised by hyperkeratosis, scaling and different degrees of inflammations affecting the entire skin. These conditions are associated with defects in protein and lipids involved in epidermal barrier formation during the cornification process, ultimately leading to an increased transepidermal water loss and dehydration (Paller, 2019; Mazereeuw‐Hautier et al., 2019; Akiyama et al., 2025; Paller et al., 2025).

Most genes implicated in hereditary forms of ichthyosis encode proteins involved in the synthesis or metabolism of lipids and structural proteins of the epidermal barrier. Alterations in these pathways represent the primary drivers of the disease phenotypes (Paller et al., 2025; Gutiérrez-Cerrajero et al., 2023; Vahlquist et al., 2018).

In some forms of hereditary ichthyosis, known as syndromic ichthyosis, the causative gene has function also outside of skin, leading to extracutaneous manifestations in gastrointestinal tract, genitourinary system and nervous system (Paller et al., 2025; Gutiérrez-Cerrajero et al., 2023; Oji et al., 2010).

Hereditary forms of non-syndromic ichthyoses can span, from the most severe forms such as harlequin ichthyosis (HI), lamellar ichthyosis (LI), congenital ichthyosis form erythroderma (CIE), epidermolytic ichthyosis (EI), recessive X-linked ichthyosis (RXLI)- to the mildest ichthyosis vulgaris (Oji et al., 2010). Regarding syndromic forms of hereditary ichthyoses, it is worth to mentioning Netherton Syndrome (NS), a rare but severe autosomal recessive disorder, with poor prognosis for affected patients due to infections and dehydration (Oji et al., 2010; Sarri et al., 2017).

HI, LI and CIE are collectively classified as autosomal recessive congenital ichthyosis (ARCI). Other genodermatoses phenotypically related to ichthyosis include autosomal dominant disorders such as Hailey-Hailey disease and Darier disease, also referred to as acantholytic disorders (Oji et al., 2010).

Treatment options are limited to palliative approaches aiming at the symptomatic relief, such as retinoid therapies used to reduce inflammation, moisturizers and keratolytic agents as topical treatment (Gutiérrez-Cerrajero et al., 2023; Rodríguez-Pazos et al., 2013).

Due to their rarity, gene therapy approaches for ichthyoses have not yet been extensively investigated.

### Autosomal recessive congenital ichthyosis

ARCI represents a heterogeneous group of autosomal recessive inherited disorders affecting different genes, with an incidence of about 1 per 100,000 general population (Koutsoukos and Bilousova, 2024).

The most frequent forms include LI and HI, often coupled with PPK (palmoplantar keratoderma), ectropion, and anhidrosis. Affected infants are frequently born as collodion babies, with enhanced perinatal mortality in severe cases (Vahlquist et al., 2018).

Lamellar ichthyosis patients are born enveloped in a collodion membrane, which disappears within the first weeks after birth, leading to the definitive phenotype of this condition. Patients show large platelike scales covering the whole body, erythroderma, ectropion, eclabium, hypoplasia of joint and nasal cartilage, scarring alopecia and PPK (Rodríguez-Pazos et al., 2013).

LI is caused by mutations in various genes (Victor and Schaffer, 2005). Among these, mutations in transglutaminase 1 (*TGM1*) gene are the most frequently identified, causing more than 30% of the LI (Russell et al., 1995). Transglutaminase catalyzes the crosslinking of loricrin and involucrin during the formation of the cornified cell envelope (Chulpanova et al., 2022), hence playing a crucial role in the formation of the cornified cell envelope in terminally differentiated keratinocytes of the stratum corneum (corneocytes) (Russell et al., 1995).

Harlequin ichthyosis represents the most severe and often life-threatening congenital form of ARCI, with an overall incidence of 1 in 300,000 births. Affected newborns are often premature and covered by thick, plate-like scales that severely distort facial features, including the lips, eyelids, ears and nostrils, due to the severe skin tightness. Mutations in the *ABCA12* gene are the most frequently identified cause of this condition (Rodríguez-Pazos et al., 2013; Shruthi et al., 2017; Kelsell et al., 2005). *ABCA12* is a member of the ATP-binding-cassette transporter family, essential, along with *ABCA3*, for alveolar surfactant lipid transport and secretion in lamellar granules (LGs), secretory granules present in upper epidermal keratinocytes. Abnormal LGs are the underlying cause of damaged skin in HI (Akiyama et al., 2005).

Netherton Syndrome is characterized by congenital ichthyosis, hair shaft abnormality and atopy, often present at birth with a collodion phenotype. NS ichthyosis is similar of that of LI (DiGiovanna and Robinson-Bostom, 2003; Sarri et al., 2017). Considering the syndromic nature of this disorder, NS patients often exhibit failure to thrive, short stature and potential intellectual disability or neurological deficits (Mocarska et al., 2025).

NS is due to mutations in *SPINK5*, the gene encoding for the serine protease inhibitor, LEKTI. LEKT1 loss of function causes epidermal protease hyperactivity, resulting in detachment of the stratum corneum, skin barrier defect, and over-desquamation (Sarri et al., 2017).

Epidermolytic ichthyosis is characterized by blistering, erosions and erythrodermic skin, with an incidence of 1:100000 to 1:400000 (Oji et al., 2010; Mendes et al., 2013; Akiyama and Shimizu, 2008). EI falls within the group of skin disorders known as keratinopathic ichthyosis (Vahlquist et al., 2018). The phenotype of EI includes skin fragility and blistering, with erythrodermic clinical manifestations at birth. The severity of these symptoms decreases with age, forming hyperkeratosis and scaling (Putra et al., 2021). EI is mostly caused by dominant mutations in the *KRT1* and *KRT10* genes (Vahlquist et al., 2018) encoding keratin 1 and keratin 10 in suprabasal keratinocytes (Kanitakis, 2002). Mutations in *KRT1* and *KRT10* lead to cytoskeleton fragility due to impairment of structural integrity of intermediate filaments (Oji et al., 2010; Chipev et al., 1992; Cheng et al., 1992). Current treatment for EI includes emollients, hydration, infection control, in addition to keratolytics to treat hyperkeratotic skin (Putra et al., 2021).

**FIGURE 1:**
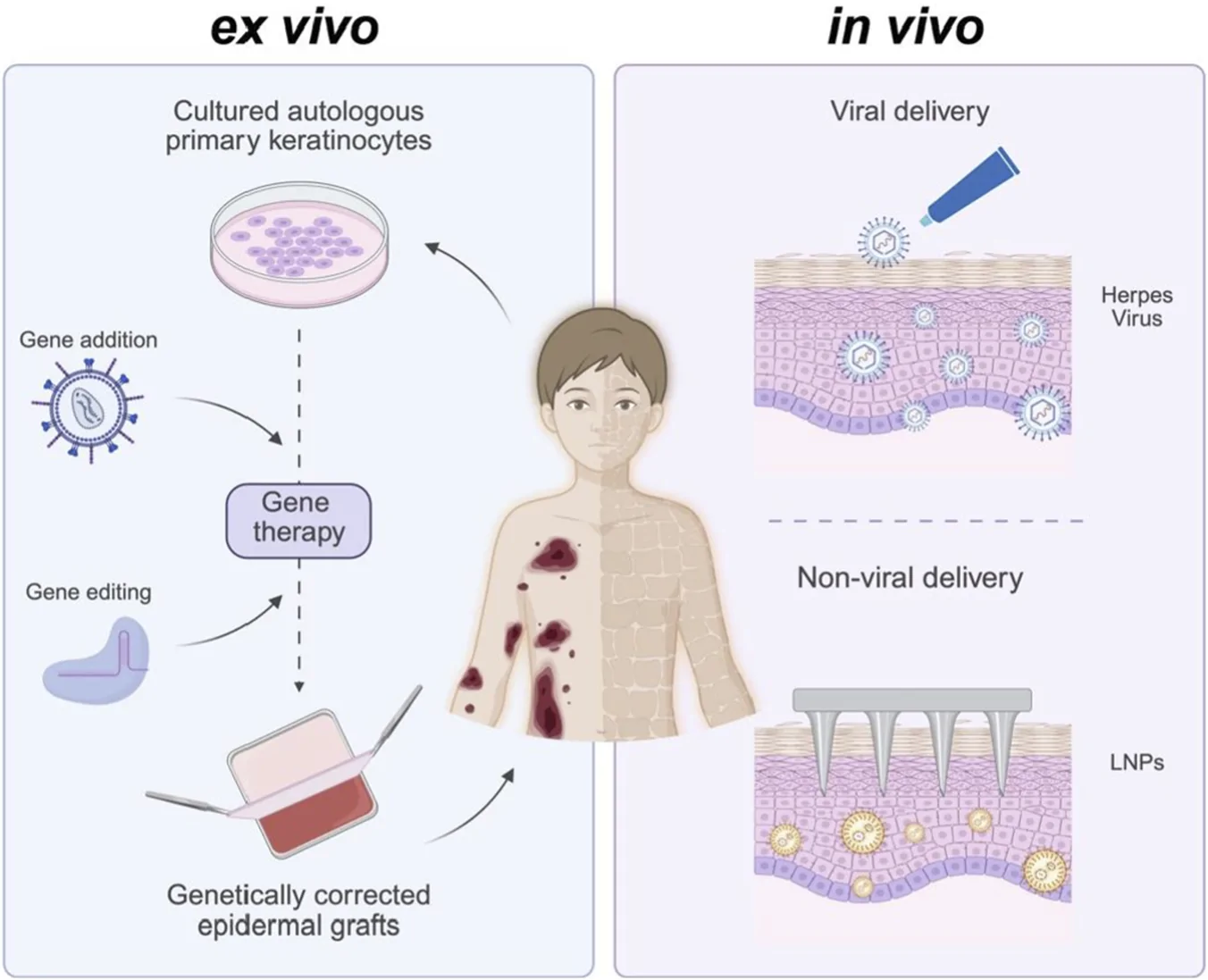
Overview of *ex vivo* and *in vivo* therapeutic strategies for inherited epidermal disorders, with a focus on current approaches for epidermolysis bullosa (left) and ichthyosis (right). *Ex vivo* gene therapy involves the genetic modification of autologous primary keratinocytes, followed by their expansion into bioengineered epidermal grafts and subsequent transplantation, with the aim of achieving long-term restoration of epidermal integrity, particularly in patients with epidermolysis bullosa. *In vivo* strategies, by contrast, rely on the direct delivery of therapeutic agents to the skin through viral or non-viral platforms, with the goal of correcting or compensating for the underlying genetic defect and restoring epidermal integrity and barrier function (Created with Biorender. com).

A general overview of available *ex vivo* and *in vivo* therapeutic strategies for inherited skin diseases is illustrated in Figure 1.

## Gene therapy approaches prior to the genome editing era

The monogenic nature of Epidermolysis Bullosa and Ichthyosis has enabled the development of multiple therapeutic strategies, such as protein replacement, allele-specific silencing and gene replacement. Those approaches can be broadly classified according to the mode of delivery (*ex vivo* or *in vivo*) and the durability of their therapeutic effect.

### *Ex vivo* cell-based therapies

*Ex vivo* therapeutic approaches for EB have primarily relied on the transplantation of allogeneic and/or autologous corrected cells. Although the dermis is considered relatively immune privileged, thereby permitting allogeneic fibroblasts transplantation (Wu et al., 1995), the highly immunogenic nature of the epidermis represents a major barrier to the engraftment of allogeneic keratinocytes (Benichou et al., 2011; Richters et al., 2005).

Early studies demonstrated that intradermally injected cultured allogeneic fibroblasts into patients with RDEB, increased collagen VII deposition at the dermal-epidermal junction (Wong et al., 2008). Building on these findings, *ex vivo* combined cell and gene therapy strategies were developed using autologous fibroblasts genetically modified with lentiviral vectors (LV) encoding *COL7A1*. Although these approaches transiently restored collagen VII deposition at the dermal-epidermal junction, long-term therapeutic benefit remained limited. Moreover, repeated injections were required owing to the limited lifespan of fibroblasts, and treatment was restricted to small and localized skin areas, limiting applicability in severe generalized RDEB (Wong et al., 2008; Lwin et al., 2019).

The structural complexity of the dermal-epidermal junction in EB, together with the lack of a defined fibroblast stem cell population in the dermis, highlighted the need for stable correction of the epidermal stem cell compartment to achieve durable tissue regeneration. This concept provided the rationale for combined *ex vivo* cell and gene therapy approaches based on genetically corrected keratinocyte stem cells. Cultured epithelial grafts generated from human keratinocytes have long been used in the treatment of extensive burns, paving the way for the development of transgenic epidermal grafts derived from genetically corrected keratinocytes (De Rosa et al., 2020; Gallico et al., 1984; Pellegrini et al., 1997; De Luca et al., 2006; De Luca et al., 2019).

The first successful combined *ex vivo* cell and gene therapy for JEB was reported in a patient with generalized intermediate JEB carrying *LAMB3* mutations. Autologous keratinocytes were transduced with a γ-retroviral vector encoding *LAMB3* cDNA and used to generate transgenic epidermal grafts (Mavilio et al., 2006). Subsequent treatment of additional patients confirmed the efficacy and safety of this approach (Hirsch et al., 2017; Bauer et al., 2017). Remarkably, this strategy proved lifesaving in a 7-year-old child presenting with near-complete epidermal loss over approximately 80% of the total body surface area. Long-term (over 10 years to date) follow-up demonstrated the persistence of a stable and fully functional epidermis and provided compelling evidence that long-term epidermal maintenance is sustained by a defined population of epidermal stem cells, identified as holoclone-forming cells (De Rosa et al., 2020; Kueckelhaus et al., 2021).

A similar strategy was subsequently applied to RDEB using autologous keratinocytes transduced with γ-retroviral vectors encoding *COL7A1*. In a phase I/II clinical trial (NCT01263379), transplantation of transgenic epidermal grafts resulted in durable wound closure and collagen VII expression in a substantial proportion of treated areas (So et al., 2022; Eichstadt et al., 2019; Siprashvili et al., 2016). These findings were later confirmed in a phase III clinical trial (NCT04227106), ultimately leading to FDA approval of ZEVASKYN (prademagene zamikeracel) in 2025 (Lee, 2025). Additional clinical studies are currently exploring skin equivalents containing both genetically corrected keratinocytes and fibroblasts (NCT04186650) (Titeux et al., 2010; Gaucher et al., 2020).

The partial success of RDEB combined *ex vivo* cell and gene therapy, if compared with the success of the ones used for JEB, can be explained by two possible reasons (i) the lower transduction efficiency of γRV-*COL7A1* with respect to γRV-*LAMB3* and (ii) the cell competition between pathological (untransduced) and transgenic keratinocytes (De et al., 2019).

In pre-clinical studies, gene delivery of *TGM1* into primary keratinocytes with restoration of TGase1 expression and functional enzymatic activity demonstrated the potential feasibility of this approach for the treatment of *TGM1*-dependent LI (Sercia et al., 2024; Choate et al., 1996). Alternative strategies explored non-viral delivery systems, including plasmid-based approaches and adenovirus-enhanced transferrin-mediated (AVET) delivery platforms in keratinocytes and organotypic skin cultures (Akiyama et al., 2005; Huber et al., 2000).

Combined *ex vivo* cell and gene therapy has also been investigated for NS caused by *SPINK5* mutations. In a phase I clinical trial, transplantation of gene-modified epidermal grafts was well tolerated and achieved transient restoration of *LEKTI* expression, although long-term correction was not maintained, (NCT01545323) (Di et al., 2019).

### *In vivo* strategies

Over the past decades, multiple *in vivo* therapeutic approaches have been developed for genodermatoses with varying degrees of success depending on the delivery platform and therapeutic cargo. However, most current strategies fail to efficiently target epidermal stem cells *in situ*, thereby limiting the durability of therapeutic benefit.

The earliest *in vivo* approaches focused on protein replacement. Recombinant collagen VII was shown to localize to the dermal-epidermal junction following topical or systemic administration in preclinical RDEB models, promoting wound healing and reducing blister formation (Woodley et al., 2013; Woodley et al., 2004; Remington et al., 2009). These findings led to clinical trials evaluating systemic recombinant collagen VII administration in RDEB patients, although no clear clinical efficacy has yet been demonstrated (NCT03752905, NCT04599881) (Bruckner et al., 2022). Similar enzyme replacement strategies were explored for lamellar ichthyosis using recombinant transglutaminase-1 delivered through liposomal or nanogel-based systems, successfully improving disease phenotypes in preclinical models, but without clinical translation to date (Plank et al., 2019; Aufenvenne et al., 2013).

Additional *in vivo* nucleic acid–based approaches have included antisense oligonucleotides (AONs), spliceosome-mediated RNA trans-splicing (SMaRT), and short interfering RNAs (siRNAs). AON-mediated exon skipping targeting *COL7A1* exon 73 was evaluated for topical treatment of RDEB and progressed to clinical testing, although the trial was terminated because of low enrolment (NCT03605069) (Bornert et al., 2021; Turczynski et al., 2012; Bremer et al., 2016). SMaRT approaches aimed to replace mutant pre-mRNA sequences with wild-type exogenous RNA fragments (Peking et al., 2019; Yang and Walsh, 2005; Berger et al., 2016; Tockner et al., 2016; Liemberger et al., 2018), whereas siRNA-based strategies were mainly explored for dominant forms of EB through intradermal or topical delivery systems (Pendaries et al., 2012; Atkinson et al., 2011; Zhou et al., 2016; Zeng et al., 2021).

A major breakthrough in *in vivo* gene addition therapy was achieved through the development of a replication-deficient herpes simplex virus type 1 (HSV-1) vector carrying *COL7A1* and formulated as a topical gel. Following successful phase I/II and phase III clinical trials (NCT03536143, NCT04491604), VYJUVEK™ (beremagene geperpavec-svdt) became the first topical gene therapy approved for DEB in both the United States and Europe (Kha et al., 2023; Gurevich et al., 2022; Guide et al., 2022; Marinkovich et al., 2025a; Marinkovich et al., 2025b).

The same HSV-1–based platform has also been investigated for *TGM1*-dependent lamellar ichthyosis and is currently undergoing clinical evaluation (Freedman et al., 2021). Similar strategies are being explored preclinically for *SPINK5*-associated Netherton syndrome (Bustos et al., 2019).

## Gene editing for genodermatoses

In recent years, gene editing has emerged as a powerful gene therapy platform for the development of permanent treatments for genetic diseases, becoming a clinical reality after the approval of the first CRISPR-based therapy, CASGEVY (Frangoul et al., 2021). Although gene editing for genodermatoses remains at a preclinical stage, ongoing efforts are directed toward developing effective gene editing strategies and achieving proof-of-concept milestones to facilitate clinical translation. This would allow to overcome major limitations of traditional gene replacement approaches, including the inherent risks of insertional mutagenesis and genotoxicity associated with integrating viral vectors, constitutive non-physiological expression of transgenes, payload constrains, and high manufacturing costs (Ottaviano and Qasim, 2025; Montini et al., 2009; Biasco et al., 2018; David and Doherty, 2017). Moreover, gene replacement strategies are less suitable for dominant conditions, in which the presence of a mutant allele exerts a pathogenic effect that cannot be corrected simply by the addition of a functional gene copy.

Conventional gene editing strategies employ programmable nucleases, including meganucleases, zinc-finger nucleases (ZFNs), transcription activator-like effector nucleases (TALENs), and the most widely adopted CRISPR/CRISPR-associated (Cas) protein system (CRISPR/Cas), to specifically recognize short stretches of nucleotides in the genome and create double-strand breaks (DSBs) (Jinek et al., 2012; Choate et al., 1996; Urnov et al., 2010; Joung and Sander, 2013). Endogenous DNA repair pathways are subsequently harnessed to introduce desired modifications at targeted genomic loci. Non-homologous end joining (NHEJ) is the predominant repair pathway, active throughout the cell cycle, and results in small insertions and deletions (INDELs) at the cleavage site when exposed to repeated nuclease cleavage (White et al., 2025; Chang et al., 2017). NHEJ-based approaches are commonly employed to disrupt the open reading frame, thereby inducing gene knock-out (KO), or to reframe genes carrying dominant negative missense or frameshift mutations, or to excise mutation-bearing exons. Alternatively, co-delivery of programmable nucleases with exogenous donor templates harbouring homology to the locus of interest engages the homology-directed repair (HDR) pathway, a high-fidelity yet less efficient pathway restricted to the S and G2 phases of the cell cycle (Liu et al., 2018), which enables accurate sequence integration (knock-in, KI) or replacement. In this manner, both dominant and recessive genetic conditions can be addressed.

The development of next-generation precision editing platforms, including base editing (BE) and prime editing (PE), has substantially broadened the capabilities of genome engineering beyond traditional nuclease-based strategies (Morren et al., 2021; De Rosa et al., 2020; Du Rand et al., 2026; Koller and Bauer, 2024; Bischof et al., 2024; Piñón Hofbauer et al., 2024). Both BE and PE technologies enable the direct and programmable correction of pathogenic variants without relying on either donor DNA templates or the induction of DSBs, as required by traditional nuclease-based strategies. Instead, they utilize nicked DNA intermediates and endogenous repair mechanisms, thereby significantly improving the safety profile and precision of genome editing. While BE systems mediate irreversible single-nucleotide conversions, making them particularly well suited for the correction of point mutations, PE platforms offer greater versatility, supporting targeted small insertions, deletions, and all possible base-to-base substitutions (Zhao et al., 2023; Gu et al., 2021; Anzalone et al., 2019). As a result, BE and PE are emerging as highly promising therapeutic strategies for both recessive and dominant genodermatoses, especially in contexts requiring precise correction of disease-causing mutations, although their clinical applicability remains challenging (Brooks et al., 2023; Apaydin et al., 2026; Osborn et al., 2020; Hong et al., 2022; Sheriff et al., 2022; Steinbeck et al., 2024; Bassons-Bascuñana et al., 2026; Fabrizi and De Rosa, 2026).

### Double-strand break dependent strategies

#### NHEJ-mediated repair therapies

For dominant-negative genetic variants, suppressing expression of the affected allele offers a straightforward and effective strategy to restore the wild-type phenotype. NHEJ-mediated indels frequently result in frameshifts within the targeted allele, typically generating premature stop codons (PTCs) that likely trigger nonsense-mediated decay of the mRNA (NMD) and subsequent knockdown of gene expression (Santiago et al., 2008). This approach has been applied to dominant-negative skin disorders, including many keratinopathies, in which mutant keratins integrate into the intermediate filament network and, under stress, cause filament collapse into aggregates, leading to cytoskeletal fragility (Luan et al., 2018; Aushev et al., 2017; Bchetnia et al., 2022; Cattaneo et al., 2024; March et al., 2019). A first non-allele specific approach was developed for *KRT5*-EBS by Aushev *et al.*, which used TALENs to target exon 1 of wild-type and mutant *KRT5* alleles thereby inducing efficient gene disruption in immortalized patient-derived EBS keratinocytes (Aushev et al., 2017). Keratinocyte clones carrying selective disruption of the mutant allele were subsequently isolated and clonally expanded for downstream characterization and functional analysis. Disruption of mutant *KRT5* allele restored IFs functionality.

Subsequent studies have increasingly shifted toward the development of mutation-specific therapeutic strategies aimed at achieving precise and durable correction while preserving endogenous gene regulation and tissue homeostasis. By employing the CRISPR/Cas9 system to selectively target the pathogenic variant c.449T>C (p.Leu150Pro) within *KRT5*, which generated a novel protospacer-adjacent motif (PAM) for SpCas9, Bchetnia and colleagues demonstrated highly efficient and mutation-specific knock-out, highlighting the great potential of this strategy (Bchetnia et al., 2022). A similar allele-specific approach was recently developed by Cattaneo *et al.*, to target a monoallelic c.475/495del21 mutation within exon 1 of *KRT14,* enabling efficient and precise discrimination of the mutant allele in primary patient-derived EBS keratinocytes (Cattaneo et al., 2024). The strategy achieved KO efficiencies of up to 94% and resulted in a full correction and preservation of epidermal stem cells, referred to as holoclone-forming cells, which play a crucial role for long-term epidermal maintenance and regeneration. For dominant DEB, Shinkuma *et al.*, targeted the dominant mutation c.8068_8084delinsGA within exon 109 of *COL7A1*, a variant known to exert a dominant-negative effect on triple helix tropocollagen formation, in induced pluripotent stem cells (iPSCs) derived from DDEB fibroblasts (Shinkuma et al., 2016). Although genetically corrected iPSCs-derived keratinocytes and fibroblasts restored collagen VII secretion, genome editing also generated truncated collagen VII species, highlighting the critical importance of precise editing design to ensure complete abrogation of pathogenic protein production while preserving normal collagen VII function.

Using a similar strategy, a non-allele specific knock-out approach for EI employed TALENs targeting exon 6 of *KRT10*, upstream of a PTC known to induce functional gene knockout (March et al., 2019). Similar to the study of Aushev *et al.*, this approach required clonal expansion, screening, and isolation of correctly edited cells, highlighting both the technical complexity of the approach and the barriers to clinical translation.

For EPPK, the CRISPR/Cas9 system was employed in an *in vivo* transgenic mouse model harbouring the c.434delAinsGGCT (p.Tyr144delinsTrpLeu) mutation within exon 1 of *KRT9* gene, which generates a novel PAM (Luan et al., 2018). Following LV-mediated delivery of the editing machinery, phenotypic mitigation was observed, marked by the normalization of abnormal epidermal differentiation and proliferation.

NHEJ-mediated restoration of the reading frame may represent a strategy for correcting frameshift variants. Statistically, one-third of expected NHEJ-induced repair outcomes should lead to reading frame restoration. This approach may be beneficial particularly for recessive frameshift mutations associated with severe forms of DEB, in which expression of mildly altered protein variants in place of null alleles could confer partial therapeutic benefit. Accordingly, the majority of gene reframing approaches to date have focused on targeting *COL7A1* (Kocher et al., 2020; Mencía et al., 2018; Takashima et al., 2019). The c.6527insC frameshift mutation in exon 80 of *COL7A1*, highly prevalent in the Spanish patient population, was successfully recovered by employing TALENs in immortalized patient-derived keratinocytes (Mencía et al., 2018). Takashima *et al.*, employed the CRISPR/Cas9 system to target a recurrent frameshift mutation, c.5819delC, within exon 70 of *COL7A1* in immortalized RDEB fibroblasts (Takashima et al., 2019). This approach resulted in functional protein restoration in approximately half of the treated cells, and collagen VII rescue within the basement membrane zone (BMZ) of collagen VII-knock-out mice treated with bulk-treated samples. To facilitate sgRNA design, *in silico* tools have been developed to predict the predominant outcomes of NHEJ repair (Chakrabarti et al., 2019). Targeting a homozygous frameshift mutation (c.6081delC) in exon 73 of *COL7A1*, Kocher *et al.*, predicted a single adenine sense-strand insertion at the target site as the dominant repair outcome, which yielded full-length collagen VII expression with only a single amino acid mismatch to the wild-type protein (Kocher et al., 2020). Functional correction was achieved in over 70% of treated primary RDEB keratinocytes, with proper localization of collagen VII within the BMZ in derived skin equivalents. However, partial intracellular retention of collagen VII within the basal keratinocyte layer persisted, potentially reflecting heterogeneity among the restored protein variants. For *COL17A1*-JEB, Bischof *et al.*, designed patient-specific Cas9-nuclease- and nickase-based targeting strategies for reframing a common homozygous deletion (c.3899_3900delCT) within exon 52 of *COL17A1* in primary JEB keratinocytes (Bischof et al., 2022). The use of paired Cas9 nickases (Cas9n) induces single-stranded DNA nicks instead of DSBs, resulting in two adjacent nicks at the target site that effectively generate a staggered DSB and greatly reducing adverse effects associated with off-target activity of wild-type Cas9. Unintended edits are limited to single nicks, which are typically repaired without detectable alterations, thereby enhancing overall safety (Ran et al., 2013). Although Bischof and colleagues reported a broader spectrum of indels generated by paired nickases, compared with wild-type Cas9, this increased editing heterogeneity translated into more homogeneous outcomes at the *COL17A1* transcript level and ultimately restored collagen XVII function (Bischof et al., 2022).

Dual sgRNA CRISPR/Cas9 system can be used to excise a region of interest located between two DSB sites. This strategy has been applied to achieve in-frame exon excision in RDEB associated gene *COL7A1*, which contains collagenous domains composed of Gly-X-Y repeat units encoded by exons that are frequently in-frame. Removal of mutation-bearing exons within these domains can produce shorter protein variants that retain partial function, thereby resulting in milder EB phenotypes (Vermeer et al., 2021). Bonafont *et al.*, employed this approach in RDEB patient keratinocytes carrying a highly prevalent frameshift mutation (c.6527insC) in exon 80 of *COL7A1* (Bonafont et al., 2019). Dual sgRNA-guided Cas9 delivered as a ribonucleoprotein complex generated DSBs upstream and downstream of exon 80, resulting in targeted exon deletion in over 80% of treated cells and subsequent expression of a shorter protein variant. Skin grafts derived from edited cells exhibited continuous collagen VII expression within the BMZ when transplanted onto immunodeficient mice. The same research group further advanced this gene-editing strategy by transitioning from *ex vivo* to *in vivo* delivery of the editing machinery *via* adenoviral vector (Ad) (García et al., 2022). A skin-humanized mouse model was generated by grafting cutaneous equivalents of patient cells, containing both keratinocytes and fibroblasts, onto immunodeficient mice. In this model, excision exon 80 resulted in collagen VII deposition at the BMZ and restoration of dermal-epidermal adhesion, demonstrating the feasibility of *in vivo* approaches to directly address blistering lesions. Recently, du Rand *et al.*, expanded the dual sgRNA CRISPR/Cas9 system for RDEB by efficiently targeting three novel *COL7A1* exons (Rodríguez-Pazos et al., 2013; Di et al., 2019; Di et al., 2019; Gu et al., 2021) harbouring pathogenic heterozygous mutations, and achieved exon deletion rates of up to 95% in primary RDEB fibroblasts and keratinocytes (Du Rand et al., 2024). Despite these promising results, the use of two distinct sgRNAs to induce adjacent DSBs in dual CRISPR/Cas9 targeting raises concerns about an increased risk of unintended outcomes, including large deletions, inversions, translocations, and insertions. Therefore, potential clinical translation would require careful design and optimization to maximize targeting specificity.

#### HDR-mediated repair therapies

Despite their high efficiency, NHEJ-mediated strategies do not enable traceless gene editing and may therefore generate protein variants with unpredictable effects. In contrast, harnessing the high-fidelity HDR pathway has been proposed as a promising approach for precise sequence integration or targeted sequence replacement. However, HDR is limited to the S/G2 phases of the cell cycle, making it less efficient than NHEJ. Consequently, early studies relied on selection steps to enrich corrected cells (Benati et al., 2018; Osborn et al., 2013; Chamorro et al., 2016; Webber et al., 2016; Hainzl et al., 2017; Kocher et al., 2017). In 2016, Webber *et al.*, reported the first application of a CRISPR/Cas9-based HDR strategy for EB (Webber et al., 2016). Primary RDEB fibroblasts carrying a mutation (4317delC) in *COL7A1* were corrected by delivering plasmids encoding the editing system and the donor repair template. A puromycin selection cassette flanked by LoxP sites was included in the template to enable selection of edited cells, followed by cre-recombinase-mediated removal, leaving a minimal LoxP footprint. iPSCs derived from the edited cells were differentiated into multiple autologous cell types, including mesenchymal stromal stem cells (MSCs), hematopoietic progenitors, and keratinocytes (Webber et al., 2016). A similar strategy was developed to correct the previously mentioned, highly recurrent c.6527insC mutation in exon 80 of *COL7A1* (Hainzl et al., 2017). Gene editing of primary RDEB keratinocytes using either SpCas9 or Cas9n, in combination with a donor plasmid containing a mRuby/puromycin selection cassette flanked by LoxP sites, resulted in phenotypic correction as demonstrated *in vitro* and *in vivo* in a xenograft mouse model. Kocher and colleagues demonstrated that double-nicking approaches represent efficient and potentially safer gene-editing strategies than wild-type Cas9 in EB (Kocher et al., 2017; Kocher et al., 2019). Co-delivery of Cas9n, paired sgRNA, and a minicircle donor vector into EBS keratinocytes corrected a dominant hotspot mutation (c.1231G>A) in exon 6 of *KRT14* associated with generalized severe EBS (Kocher et al., 2017). Following antibiotic selection, recombination efficiency exceeded 30%, yielding a *KRT14* correction efficiency of over 16%. A similar selection-based double-nicking approach was used to target a splice-site mutation (c.425A > G) within exon 3 of *COL7A1*, accomplishing remarkably high HDR frequencies of 89% while highly limiting unwanted repair outcomes (Kocher et al., 2019).

Fluorescent-based cell sorting was used by Jacków and colleagues to enrich for edited iPSCs derived from primary RDEB fibroblasts from two patients with different mutational backgrounds (patient 1: c.2470insG/c.2470insG; patient 2: c.2470insG/c.3948insT) (Jacków et al., 2019). iPSCs were co-electroporated with the CRISPR/Cas9 system as ribonucleoprotein (RNP) complex, single-stranded oligodeoxynucleotide (ssODN) as donor templates and a plasmid encoding green fluorescent protein (GFP) to enable selection for transfected cells. Corrected iPSCs were subsequently differentiated into keratinocytes and fibroblasts to generate skin equivalents (SE), which demonstrated restoration of collagen VII expression and skin integrity when grafted onto immunodeficient mice (Jacków et al., 2019). Recently, Neumayer *et al.*, further advanced this approach by establishing a current Good Manufacturing Practice (cGMP)-protocol that integrates gene editing and iPSC reprogramming into a single manufacturing step (Neumayer et al., 2023). The process involved transfecting RDEB primary fibroblasts with high-fidelity Cas9 (hfCas9) RNPs, ssODN donor templates, and reprogramming factors. Corrected iPSCs were obtained within 4 weeks and subjected to generate organotypic skin grafts composed of basal keratinocytes, dermal fibroblasts and melanocytes, faithfully resembling the composition of physiological tissues. Following transplantation onto immunodeficient mice, the grafts developed a stratified epidermis expressing collagen VII at the BMZ. Although iPSCs circumvent constraints inherent to primary epidermal cell cultures, including limited clonal expansion and reduced manipulability, the long-term skin regenerative capacity and safety profile of human keratinocytes and fibroblasts derived from iPSCs have yet to be established.

HDR-mediated gene editing has also enabled insertion of a quasi-complete *LAMB3* cDNA at its endogenous locus, potentially addressing multiple JEB-causing mutations (Benati et al., 2018). Immortalized JEB keratinocytes were co-transduced with an Ad coding for SpCas9 and gRNA, and non-integrating lentiviral vector (IDLV) bearing the therapeutic donor template. Despite low HDR efficiency in the absence of selection (<0.5%), the authors leveraged the *in vitro* adhesion advantage conferred by laminin 332 production to positively select *LAMB3*-expressing keratinocytes, resulting in 17% correction. Grafting of genetically corrected skin equivalents onto immunodeficient mice resulted in restoration of the dermal-epidermal junction.

Recent advances in gene-editing technologies, encompassing the use of the CRISPR/Cas9 system as RNPs and optimized donor templates delivered either by co-electroporation together with RNPs or via adeno associated viral vector (AAV) transduction, have significantly enhanced HDR-mediated editing efficiencies. These improvements have enabled to avoid antibiotic- or fluorescence-based enrichment strategies, which can be cumbersome and cytotoxic, and may leave LoxP footprints at the targeted locus, thereby hindering clinical translation. In 2018, Izmiryan *et al.*, presented a selection-free CRISPR/Cas9-based *COL7A1* editing approach to treat primary RDEB fibroblasts and keratinocytes harbouring a null mutation (c.189delG) in exon 2 (Izmiryan et al., 2018). IDLV-mediated delivery of the gRNA together with the donor template yielded 11% and 15.7% correction of *COL7A1* transcripts in primary keratinocytes and fibroblasts, respectively, and up to 19% in gene-corrected skin grafts. Functional rescue of collagen VII expression and anchoring fibrils formation at the dermal-epidermal junction was observed in a murine xenograft model (Izmiryan et al., 2018). To mitigate risks of off-target Cas9 nuclease activity resulting from prolonged expression within edited cells, recent strategies delivered the CRISPR/Cas9 system as RNP complexes, characterized by short intracellular half-life. Electroporation of paired Cas9 nickases together with ssODN donor templates achieved correction efficiencies of 21% and 10% in RDEB patient-derived keratinocytes and fibroblasts, respectively, carrying the splice site-mutation c.425A>G in *COL7A1* (Kocher et al., 2021). This resulted in full-length collagen VII restoration and its accurate, albeit irregular, deposition within skin equivalents, suggesting a need for improved HDR efficiency. Petković and colleagues developed a similar approach to correct the frameshift mutation c.3899_3900delCT in primary *COL17A1*-JEB keratinocytes, yielding impressive HDR-based correction efficiencies of up to ∼38% using high-fidelity Cas9 (HiFiCas9), as compared to ∼18% when employing paired Cas9 nickases (Petković et al., 2022). Engineered SEs exhibited accurate and nearly continuous deposition of collagen XVII along the dermal-epidermal junction. Recently, du Rand *et al.*, reported up to 54% precise correction of the *LAMB3* c.1903C>T mutation by combining dual Cas9n with modified ssODN donor templates in primary JEB keratinocytes (du Rand et al., 2025). This led to the restoration of laminin-332 secretion, rescuing JEB keratinocyte adhesion *in vitro* and enabling accurate laminin-332 deposition at the BMZ in engineered SEs. In one study, Bonafont at all. leveraged AAV-based donor template delivery in combination with CRISPR/Cas9 RNPs to target two different RDEB-causing variants in exon 79 (c.6501G>T) and 80 (c.6527insC) of *COL7A1* in primary patient-derived keratinocytes, achieving similar correction frequencies of up to 50% (Bonafont et al., 2021). Two alternative donor templates were evaluated, featuring either symmetric arms spanning exons 74–84 or asymmetric arms spanning exons 77–88, yielding comparable results. As these regions encompass a substantial portion of the *COL7A1* gene, where approximately 35% of DEB-associated mutations are located, the authors proposed that a single donor template could be used to simultaneously target multiple RDEB-causing mutations (Van den Akker et al., 2011). Grafting of gene-corrected SEs onto immunodeficient mice showed normal skin architecture with continuous collagen VII deposition at the BMZ for up to 12 weeks. Recently, Berhault and colleagues achieved highly efficient correction rate of up to 58% in primary keratinocytes and fibroblasts derived from RDEB patients harbouring the c.6508C>T mutation in exon 80 of *COL7A1* by delivering RNPs and ssODN templates (Berthault et al., 2024). Transplantation of corrected SEs onto nude mice resulted in long-term re-expression and correct localization of functional collagen VII at the dermal-epidermal junction. Notably, the highest editing efficiency reported to date for EB, reaching up to 75%, was recently achieved for *COL7A1* by co-delivering Cas9 RNPs with short single-stranded DNA (ssDNA) repair templates in combination with the small molecule M3814, a potent inhibitor of DNA-PKcs, a key component of the NHEJ pathway (Hunt et al., 2025). This strategy was employed to target three different mutations (c.2278_2279del, c.8698_8708del, c.3551-3T>G) in RDEB fibroblasts and keratinocytes, yielding HDR efficiencies of up to 32% and 75%, respectively. Re-expression of collagen VII protein at the dermal-epidermal junction was observed *in vitro* engineered Ses (Hunt et al., 2025).

Despite major advances in HDR-mediated gene editing for EB have led to remarkable editing efficiencies and compelling proof-of-concept results, no evidence has yet demonstrated effective targeting of epidermal stem cells, a prerequisite for the success of long-term gene therapy approaches. Furthermore, DSB-related genotoxicity, general low efficiency, reliance on exogenous templates, and challenges with *in vivo* delivery, all hinder the clinical translation of HDR-based approaches.

#### Double-strand break independent strategies

DSB-independent genome editing strategies, including BE and PE, are rapidly reshaping the therapeutic landscape for genodermatoses by enabling precise correction of pathogenic variants without the risks associated with DSB-induced DNA damage and error-prone repair (Brooks et al., 2023; Anzalone et al., 2019; Xu et al., 2024; Eid et al., 2018). Base editors combine a catalytically impaired Cas9 (dCas9 or Cas9 nickase) with a nucleotide deaminase to mediate programmable transition mutations within a defined editing window. In particular, cytidine base editors (CBEs) convert C•G to T•A, while adenine base editors (ABEs) convert A•T to G•C without requiring donor DNA templates (Gu et al., 2021). More recent generations, including glycosylase base editors and dual-function editors, have expanded the scope to include selected transversion mutations and reduced bystander activity, although editing outcomes remain highly sequence- and context-dependent (Wang et al., 2024; Tao et al., 2026). In contrast, PE broadens the spectrum of editable variants by coupling a Cas9 nickase to a reverse transcriptase and a PE guide RNA (pegRNA), enabling all base substitutions as well as small insertions and deletions through a “search-and-replace” mechanism independent of DSBs or donor templates (Zhao et al., 2023; Anzalone et al., 2019). Advanced PE systems, such as PE3 and PE5, incorporate additional nicking strategies or mismatch repair modulation to enhance efficiency, albeit sometimes at the cost of increased indel formation (Zhao et al., 2023; Lee et al., 2025). From a comparative perspective, BE currently offers higher efficiency and simpler design for transition mutations, whereas PE provides greater versatility for complex or non-transition variants, making these approaches complementary rather than interchangeable in the context of precision dermatology (Gao et al., 2025).

These technologies are particularly relevant for EB, ichthyoses, and other genodermatoses, in which a substantial proportion of disease-causing variants are single-nucleotide substitutions.

In RDEB, caused by mutations in *COL7A1*, both BE and PE have demonstrated effective correction of disease-causing variants in patient-derived keratinocytes, fibroblasts, and iPSCs, leading to restoration of collagen VII and formation of anchoring fibrils in 3D skin equivalents. Osborn *et al.*, demonstrated that ABE efficiently corrected pathogenic alleles in primary RDEB fibroblasts and fibroblast-derived iPSCs from two patients: one carrying a homozygous c.553C>T (R185X) mutation and another with compound heterozygous c.1573C>T (R525X) and c.2005C>T (R669X) mutations (Osborn et al., 2020). Correction restored *COL7A1* mRNA and collagen VII protein expression and enabled the generation of skin tissue with proper collagen VII deposition in a mouse teratoma model (Osborn et al., 2020). Similarly, Hong *et al.*, applied both BE (c.3631C>T (Q1211X)) and PE (c.3631C>T (Q1211X) and c.2005C>T (R669X)) to patient fibroblasts, and transplantation of edited cells into immunodeficient mice resulted in detectable collagen VII at the dermal-epidermal junction and formation of functional anchoring fibrils (Hong et al., 2022).

Using an evolved ABE8e, Sheriff *et al.*, achieved very high editing efficiency (∼94%) of the recurrent c.5047C>T mutation in patient fibroblasts, which led to robust restoration of *COL7A1* expression and deposition of collagen VII in 3D skin constructs (Sheriff et al., 2022). More recently, another study confirmed that ABE8e editing of patient-derived keratinocytes and fibroblasts cell lines (immortalized with E6/E7) derived from one patient harbouring a homozygous mutation in exon 72 (c.5932C>T, p. Arg1978Ter), and a second patient carrying a heterozygous mutation in exon 94 (c.7249C>T, p. Gln2417Ter), along with a second mutation in exon 80 (c.6527dupC, p. Gly2177Trp, fs∗113)), achieved >90% correction efficiency, with functional recovery of collagen VII in skin equivalents and minimal off-target effects, highlighting the translational potential of these approaches (Bassons-Bascuñana et al., 2026; Fabrizi and De Rosa, 2026).

Steinbeck *et al.*, demonstrated that twin PE using PEmax and the evolved PE6 system, together with dual pegRNAs targeting *COL7A1* exon 5, enabled efficient exon excision and reconstitution across patient-derived fibroblasts, keratinocytes, and iPSCs (Steinbeck et al., 2024). Editing efficiencies were generally in the ∼20–50% range in patient-derived cells, reaching up to ∼50–70% under optimized conditions depending on editor configuration, pegRNA design, and delivery format. Beyond restoring type VII collagen expression, this study further showed that a programmable twin PE strategy can install recombinase-compatible landing sites for precise sequence replacement, extending its utility from exon skipping to targeted exon rewriting (Steinbeck et al., 2024). Importantly, this approach achieved relatively low indel formation and minimal predicted off-target effects, and restored functional collagen VII deposition in engineered skin models, supporting its potential as a broadly applicable and clinically relevant platform for RDEB correction (Steinbeck et al., 2024).

In contrast to RDEB, where BE and PE approaches are increasingly being explored, the application of DSB-independent editing technologies to JEB and EBS remains relatively scarce.

Early BE studies have begun to explore the potential of precise genomic correction for inherited ichthyoses, particularly for ARCI caused by pathogenic variants in *TGM1*. Recent preclinical work demonstrated that a CBE delivered via topical lipid nanoparticle (LNP) formulations can correct a common ARCI-associated *TGM1* c.877-2A>G splice-site mutation in on organotypic skin models (from immortalized keratinocytes and fibroblasts) human skin models and partially restore transglutaminase-1 (TG1) enzymatic activity *in situ* (∼30% restoration), highlighting the feasibility of editing pathogenic alleles in clinically relevant epidermal contexts (Apaydin et al., 2026). Although this work underscores the therapeutic potential of BE in ARCI by achieving functional enzyme recovery in organotypic skin models, robust *in vivo* validation, particularly in clinically relevant animal models (xenotransplantation) or human subjects, remains limited. This gap highlights the challenges in translating experimental successes with BE into therapeutic readiness for ichthyoses, a challenge that mirrors broader gaps in the field of therapeutic gene editing for genodermatoses more generally.

All therapeutic strategies for genodermatoses discussed in this Review are summarized in Table 1.

**TABLE 1:** Summary of therapeutic strategies for genodermatoses.

Gene therapy approaches prior to the genome editing era					
Strategy	Disease	Gene	Vector/Strategy	Delivery (*ex vivo/in vivo*)	References
Gene addition	RDEB	*COL7A1*	LV	*Ex vivo* (primary fibroblast)	Lwin et al. (2019) NCT02810951, NCT02493816, NCT04213261
	JEB	*LAMB3*	γRV	*Ex vivo* (primary keratinocytes)	Bauer et al. (2017), Mavilio et al. (2006), Hirsch et al. (2017), Siprashvili et al. (2016)
	RDEB	*COL7A1*	γRV	*Ex vivo* (primary keratinocytes)	Eichstadt et al. (2019), Titeux et al. (2010) NCT01263379, NCT04227106, NCT04186650
	LI	*TGM1*	RV	*Ex vivo* (primary keratinocytes)	Choate et al. (1996)
	LI	*TGM1*	SIN-γRV	*Ex vivo* (primary keratinocytes)	Sercia et al. (2024)
	HI	*ABCA12*	Plasmids	*Ex vivo* (primary keratinocytes)	Akiyama et al. (2005); Huber et al. (2000)
	LI	*TGM1*	AVET system with plasmid DNA	*Ex vivo* (primary keratinocytes and organotypic cultures)	Huber et al. (2000)
	NS	*SPINK5*	LV	*Ex vivo* (primary keratinocytes)	Di et al. (2019) NCT01545323
	RDEB	*COL7A1*	HSV-1	*In vivo* (topical)	Gurevich et al. (2022) NCT03536143 Guide et al. (2022) NCT04491604
	LI	*TGM1*	HSV-1	*In vivo* (topical)	Freedman et al. (2021) NCT04047732
Protein therapy	RDEB	*COL7A1*	Recombinant protein	*In vivo* (intradermic or systemic)	Woodley et al. (2013); Remington et al. (2009); Woodley et al. (2004) NCT03752905, NCT04599881
	LI	*TGM1*	Recombinant protein	*In vivo*/*Ex vivo* (mouse model/skin equivalents)	Aufenvenne et al. (2013); Plank et al. (2019)
RNA approaches	RDEB	*COL7A1*	AON exon 73	*Ex vivo* (primary keratinocytes and fibroblasts)	Bremer et al. (2016); Bornert et al. (2021) NCT03605069
	RDEB	*COL7A1*	SMaRT	*Ex vivo* (RDEB patient-derived cell line)	Tockner et al. (2016)
	RDEB	*COL7A1*	siRNA	*In vivo* (mouse model)	Zeng et al. (2021); Zhou et al. (2016)
	EBS	*KRT14*	SMaRT	*Ex vivo* (EBDM-1 cell line)	Peking et al. (2019)

Double-strand break dependent strategies					
Strategy	Disease	Gene	Mutation	Delivery (*ex vivo/in vivo*)	References
NHEJ (knock-out)	EBS	*KRT5*	Non-allele specific	*Ex vivo* (immortalized keratinocytes)	Aushev et al. (2017)
	EBS	*KRT5*	c.449T>C	*Ex vivo* (primary keratinocytes)	Bchetnia et al. (2022)
	EBS	*KRT14*	c.475/495del21	*Ex vivo* (primary keratinocytes)	Cattaneo et al. (2024)
	DEB	*COL7A1*	c.8068_8084delinsGA	*Ex vivo* (iPSCs)	Shinkuma et al. (2016)
	EI	*KRT10*	Non-allele specific	*Ex vivo* (immortalized and primary keratinocytes)	March et al. (2019)
	EPPK	*KRT9*	c.434delAinsGGCT	*In vivo* (LV in humanized mouse model)	Luan et al. (2018)
NHEJ (reframing)	DEB	*COL7A1*	c.6527insC	*Ex vivo* (immortalized keratinocytes)	Mencía et al. (2018)
	DEB	*COL7A1*	c.5819delC	*Ex vivo* (immortalized and primary fibroblasts)	Takashima et al. (2019)
	DEB	*COL7A1*	c.6081delC	*Ex vivo* (primary keratinocytes)	Kocher et al. (2020)
	JEB	*COL17A1*	c.3899_3900delCT	*Ex vivo* (primary keratinocytes)	Bischof et al. (2022)
NHEJ (in-frame exon excision)	RDEB	*COL7A1*	c.6527insC	*Ex vivo* (primary keratinocytes)	Bonafont et al. (2019)
	RDEB	*COL7A1*	c.6527insC	*In vivo* (AdV in humanized mouse model)	Du Rand et al. (2024)
	RDEB	*COL7A1*	c.3840del; c.5720_5721delGAinsAT; c.8065G>A	*Ex vivo* (primary keratinocytes and fibroblasts)	du Rand et al. (2024)
HDR	RDEB	*COL7A1*	4317delC	*Ex vivo* (iPSCs)	Webber et al. (2016)
	RDEB	*COL7A1*	c.6527insC	*Ex vivo* (immortalized keratinocytes)	Hainzl et al. (2017)
	EBS	*KRT14*	c.1231G>A	*Ex vivo* (primary keratinocytes)	Kocher et al. (2017)
	RDEB	*COL7A1*	c.425A > G	*Ex vivo* (immortalized keratinocytes)	Kocher et al. (2019)
	RDEB	*COL7A1*	c.2470insG; c.3948insT	*Ex vivo* (iPSCs)	Jacków et al. (2019)
	RDEB	*COL7A1*	c.7485+5G>A; c.6781C>T	*Ex vivo* (iPSCs)	Neumayer et al. (2023)
	JEB	*LAMB3*	c.1945dupG; c.1903C > T	*Ex vivo* (immortalized keratinocytes)	Benati et al. (2018)
	RDEB	*COL7A1*	c.189delG	*Ex vivo* (primary keratinocytes and fibroblasts)	Izmiryan et al. (2018)
	RDEB	*COL7A1*	c.425A>G	*Ex vivo* (primary keratinocytes and fibroblasts)	Kocher et al. (2021)
	JEB	*COL17A1*	c.3899_3900delCT	*Ex vivo* (primary keratinocytes)	Petković et al. (2022)
	JEB	*LAMB3*	c.1903C>T	*Ex vivo* (primary keratinocytes)	Du Rand et al. (2025)
	RDEB	*COL7A1*	c.6501G>T; c.6527insC	*Ex vivo* (primary keratinocytes)	Bonafont et al. (2021)
	RDEB	*COL7A1*	c.6508C>T	*Ex vivo* (primary keratinocytes and fibroblasts)	Berthault et al. (2024)
	RDEB	*COL7A1*	c.2278_2279del; c.8698_8708del; c.3551-3T>G	*Ex vivo* (primary keratinocytes and fibroblasts)	Hunt et al. (2025)

Double-strand break independent strategies					
Strategy	Disease	Gene	Mutation	Delivery (*ex vivo/in vivo*)	References
ABE	RDEB	*COL7A1*	c.553C>T; c.1573C>T	*Ex vivo* (primary fibroblasts, iPSCs)	Osborn et al. (2020)
BE and PE	RDEB	*COL7A1*	c.3631C > T (BE); c.3631C>T and c.2005C>T (PE)	*Ex vivo* (primary fibroblasts)	Hong et al. (2022)
Evolved ABE8e	RDEB	*COL7A1*	c.5047C>T	*Ex vivo* (primary fibroblasts)	Sheriff et al. (2022)
ABE8e	RDEB	*COL7A1*	c.5932C>T; c.7249C>T and c.6527dupC	*Ex vivo* (immortalized keratinocytes and fibroblasts)	(Bassons-Bascuñana et al.; Fabrizi and De Rosa, 2026)
Twin PE	RDEB	*COL7A1*	Mutation-agnostic exon skipping/reinsertion strategy	*Ex vivo* (primary keratinocytes and fibroblasts, iPSCs)	Steinbeck et al. (2024)
CBE	ARCI	*TGM1*	c.877-2A>G	LNP *in situ* delivery on organotypic skin models from immortalized keratinocytes and fibroblasts	Apaydin et al. (2026)

Abbreviations: NHEJ: Non-Homologous End Joining; HDR: Homology-Directed Repair; BE: Base Editing; PE: Prime Editing; ABE: Adenine Base Editor; CBE: Cytosine Base Editor; RDEB: Recessive Dystrophic Epidermolysis Bullosa; JEB: Junctional Epidermolysis Bullosa; LI: Lamellar Ichthyosis; HI: Harlequin Ichthyosis; NS: Netherton Syndrome; EBS: Epidermolysis Bullosa Simplex; DEB: Dystrophic Epidermolysis Bullosa; EI: Epidermolytic Ichthyosis; EPPK: Epidermolytic Palmoplantar Keratoderma; ARCI: Autosomal Recessive Congenital Ichthyosis; LV: Lentiviral Vector; RV: Retroviral Vector; SIN-γRV: Self-Inactivating Gamma-Retroviral Vector; AdV: Adenoviral Vector; AVET system: Amplification Vector Expression Technology system; HSV-1: Herpes Simplex Virus Type 1; AON: Antisense oligonucleotide; SMaRT: Spliceosome-mediated mRNA trans-splicing; siRNA: small interfering RNA; LNP: Lipid Nanoparticle; iPSCs: Induced Pluripotent Stem Cells.

## Conclusion and perspectives

Inherited genodermatoses, including EB and ichthyoses, represent rare monogenic skin disorders with well-characterized molecular basis (Koller and Bauer, 2021). These disorders have emerged as highly valuable paradigms for gene therapy and precision medicine, owing to the accessibility of the skin and the possibility to isolate, propagate and genetically manipulate epidermal stem cells *ex vivo* (De Rosa et al., 2014; De Rosa et al., 2020).

Most of the gene therapy strategies proposed for genodermatoses have in fact been developed in an *ex vivo* setting, providing compelling evidence for long-term functional skin restoration (Hirsch et al., 2017).

Integrating γ-retroviral and lentiviral vectors remain the most widely used platforms for gene delivery in *ex vivo* gene therapy of genodermatoses. Integrating vectors enable stable and long-term transgene expression in epidermal stem cells, allowing the permanent restoration of a functional epidermis (De Rosa et al., 2014; Hirsch et al., 2017). Although no oncogenic events have been reported in any of the trials and studies (De Rosa et al., 2014; Hirsch et al., 2017; Mavilio et al., 2006; Kueckelhaus et al., 2021; So et al., 2022; Eichstadt et al., 2019; Siprashvili et al., 2016), random genomic integration of such vectors raises concerns about insertional mutagenesis, as observed in early gene therapy trials for hematopoietic diseases (Hacein-Bey-Abina et al., 2008). The development of self-inactivating vectors has greatly improved safety, but long-term follow-up remains necessary.

*Ex vivo* therapies require specialized GMP facilities, extensive cell manipulation, surgical procedures and highly trained personnel, all of which limit their scalability and affordability. For these reasons, gene therapy strategies are moving towards *in vivo* approaches offering a less invasive and potentially more scalable alternative. The approval of VYJUVEK™ (beremagene geperpavec), the first topical HSV-1-based gene therapy for RDEB, represents a significant milestone in this direction, which has been followed by similar approaches in *TGM1*-dependent LI (NCT04047732) and *SPINK5*-dependent Netherton syndrome (Kha et al., 2023; Bustos et al., 2019).

However, the non-integrating nature of HSV-1 results in a transient gene expression, which requires repeated, perhaps lifelong administration and raises concerns regarding long-term cost, patient compliance, and cumulative safety (Kha et al., 2023). In fact, this approach might not prove suitable for severe generalized forms of EB affecting the majority of the body surface. In this latter clinical scenario, *ex vivo* approaches may take advantage of the possibility of cultivating a large number of transgenic grafts containing an adequate number of stably transduced epidermal stem cells. Such cultures, when engrafted, can permanently restore a functional epidermis (Hirsch et al., 2017), provided a proper wound bed preparation and management of the chronic inflammation often affecting severe conditions such as generalized RDEB.

In fact, one of the most critical and often underestimated challenge across all gene therapy strategies for genodermatoses remains the effective targeting and long-term maintenance of epidermal stem cells. Durable clinical correction not only requires successful and proper gene editing but also the preservation of stem cell identity, proliferative capacity, and competitive fitness within the native tissue environment.

In particular, the efficiency of the newly proposed gene editing strategies remains suboptimal in epidermal stem cells (as compared to immortalized or pluripotent stem cells), and their impact on stem cell behaviour is not yet fully understood (Brooks et al., 2023).

Another critical limitation for DSB-dependent gene editing strategies is due to the aberrant activity of programmable nucleases. Unintended off-target genomic cleavage poses genotoxic risks and potential tumorigenicity, hindering the clinical translation of CRISPR/Cas-based gene therapies. To mitigate off-target activity, the systematic selection and rigorous evaluation of the different gRNAs candidates represent a critical step. To date there are several *in silico* tools for gRNAs design and off-target prediction, including CRISPRon, CHOPCHOP, Cas-OFFinder and CRISPRoff, which aim at maximizing on-target activity while minimizing off-target risk (Anthon et al., 2022; Labun et al., 2019; Bae et al., 2014).

Moreover, multiple orthogonal and comprehensive *in vitro* (e.g.,: CIRCLE-seq, CHANGE-seq) and *in cellular* based experimental approaches (e.g.,: GUIDE-seq, long-read sequencing, ddPCR-based methods, single-cell RNA sequencing (scRNA-seq), CAST-seq) are nowadays used to robustly delineate both the off-target landscape and on-target aberration events, thereby defining the safety profile of a given DSB-dependent gene editing strategy (Turchiano et al., 2021; Tsai et al., 2017; Lazzarotto et al., 2020; Tsai et al., 2015)^.^ Furthermore, high-fidelity Cas9 variants or paired Cas9 nickases are now being employed to further improve the overall safety via induction of DSB-repair pathways, even though dual Cas9n systems have still shown on-target chromosomal aberrations (Bischof et al., 2022; Ran et al., 2013; Kocher et al., 2021; Klermund et al., 2024; Kleinstiver et al., 2016; Cho et al., 2014).

Additional safety concerns in relation to DSB-dependent gene editing strategies arise from the use of NHEJ inhibitors used to increase the efficiency of HDR-mediated editing strategies. Inhibition of DNA-PKcs has in fact been associated with large-scale genomic alterations, including kilobase- and megabase-scale deletions, chromosome arm loss and translocations, underscoring the need for rigorous evaluation of whether the use of NHEJ-inhibitors might in fact pose additional risks on genomic stability that may ultimately limit the translation into clinic (Cullot et al., 2025; Schimmel et al., 2023; Wimberger et al., 2023).

Safer gene editing approaches include the use of BE and PE, which offer safety advantages over wild-type Cas9 nuclease, largely due to their independence from DSBs. In spite of this, their clinical translation is still limited by several technical constraints. Both BE and PE, while avoiding DSBs, can in fact introduce unintended modifications, including bystander edits within the editing window, low-frequency indels, and off-target deamination events at both DNA and RNA levels (Grünewald et al., 2019; Park and Beal, 2019). These off-targets are minimized in next-generation high-fidelity BE editors, but they are not completely resolved (Grünewald et al., 2019; Park and Beal, 2019; Kantor et al., 2020). On the other hand, despite PE approaches offers greater versatility and generally lower off-target activity than both BE and traditional nucleases, because the exact sequence is templated by the pegRNA, they still produce small indels and shows variable efficiency dependent on pegRNA design, cellular context, and DNA repair dynamics (Brooks et al., 2025). Due to these limitations, no clinical trials have yet evaluated BE and PE technologies in patients with EB or ichthyosis, highlighting the gap between promising preclinical data and real-world therapeutic application (Brooks et al., 2023; Apaydin et al., 2026; Osborn et al., 2020; Hong et al., 2022; Sheriff et al., 2022; Steinbeck et al., 2024; Bassons-Bascuñana et al., 2026; Fabrizi and De Rosa, 2026).

Apart from choosing the correct genetic toolbox between DSB-dependent and -independent approaches, a major bottleneck for clinical translation is the safe and efficient delivery of these relatively large editing systems to the epidermal stem cell compartment both *ex vivo* and *in vivo*.

Non-viral approaches, including lipid nanoparticles-mediated delivery, electroporation, and topical formulations, offers safer and more attractive alternatives but still require substantial optimization to improve tissue targeting, cargo protection, immune tolerance, and scalability for therapeutic use (Hong et al., 2022; Lee et al., 2025; Masarwy et al., 2024).

Beyond the choice of the delivery vehicle itself, increasing attention is also being devoted to aimed at enhancing cellular uptake, intracellular trafficking, endosomal escape and tissue-specific targeting of therapeutic cargo. Although these approached have shown promising results in other tissues, their application to cutaneous gene therapy remains largely unexplored (Khalilzad et al., 2025). Further investigation is therefore required to determine their efficacy and safety in the skin and to assess their potential to overcome current delivery barriers and facilitate clinical translation.

Overall, the choice of delivery platform reflects a critical balance between durability and safety, further emphasizing that the successful translation of gene therapies for genodermatoses will not only depend on efficacy but also on the development of safer and more efficient delivery strategies.

Taken together, these observations highlight that the currently available therapeutic platforms should be viewed as complementary rather than competing strategies. Gene replacement therapies currently represent the most clinically mature approaches and provide the strongest evidence for durable restoration of functional skin, particularly when applied *ex vivo* (De Rosa et al., 2014; Hirsch et al., 2017; De Rosa et al., 2020; Mavilio et al., 2006; Bauer et al., 2017; Kueckelhaus et al., 2021; So et al., 2022; Eichstadt et al., 2019; Siprashvili et al., 2016; Lee, 2025; Titeux et al., 2010; Gaucher et al., 2020). However, their broader clinical application remains constrained by complex manufacturing procedures, the need for surgical intervention, non-physiological transgene expression, and, in the case of integrating vectors, the potential risk of insertional mutagenesis (Hacein-Bey-Abina et al., 2008; De Luca and Cossu, 2023). Conversely, gene editing strategies enable precise correction of the endogenous mutant allele while preserving physiological gene regulation (Du Rand et al., 2026). Although DSB-dependent approaches continue to raise concerns regarding off-target effects and genomic instability, DSB-independent technologies, including BE and PE, offer an improved safety profile by avoiding double-strand DNA breaks (Zhao et al., 2023; Gu et al., 2021; Anzalone et al., 2019; Xu et al., 2024; Eid et al., 2018; Wang et al., 2024; Tao et al., 2026; Lee et al., 2025; Gao et al., 2025; Cho et al., 2014). However, their clinical translation is still constrained by editor-specific limitations, including PAM availability, bystander editing, variable editing efficiency, and the absence of clinical validation in genodermatoses (Apaydin et al., 2026; Osborn et al., 2020; Hong et al., 2022; Sheriff et al., 2022; Steinbeck et al., 2024; Bassons-Bascuñana et al., 2026; Fabrizi and De Rosa, 2026; Wang et al., 2024; Tao et al., 2026; Lee et al., 2025; Gao et al., 2025; Grünewald et al., 2019; Park and Beal, 2019; Kantor et al., 2020). Importantly, regardless of the editing strategy employed, efficient delivery to long-lived epidermal stem cells remains a common bottleneck that ultimately determines therapeutic durability and clinical applicability (De Rosa et al., 2014; Hirsch et al., 2017; De Rosa et al., 2020; Kueckelhaus et al., 2021). Therefore, the choice of therapeutic platform should be guided not only by the underlying mutation but also by disease severity, delivery feasibility, and the balance between efficacy, safety, and long-term durability.

Looking ahead, the future of gene- and cell-based therapies for genodermatoses will likely depend on the integration of complementary strategies rather than the dominance of a single approach. Advances in gene editing technologies, including improved BE and PE systems with enhanced precision and efficiency, together with innovations in delivery platforms such as LNPs may enable effective and less invasive *in vivo* therapies. At the same time, continued progress in stem cell biology and skin bioengineering will refine *ex vivo* approaches, potentially improving their scalability and robustness.

Beyond biological and technological considerations, also economic and regulatory challenges significantly influence the trajectory of therapeutic development. Advanced Therapy Medicinal Products (ATMPs) are associated with high manufacturing costs, complex regulatory pathways, and stringent quality requirements (De Luca and Cossu, 2023; Pellegrini et al., 2018; Farkas et al., 2017; Pellegrini et al., 2016). These challenges are exacerbated by the rarity and heterogeneity of genodermatoses, which limit the size of treatable patient populations and complicate the design of large-scale clinical trials. Moreover, the increasing shift toward mutation-specific or even patient-specific therapies, while scientifically justified, raises concerns regarding sustainability, reimbursement, and equitable access (Francisco et al., 2025). For these reasons, the development of platform technologies targeting recurrent mutations or broader genomic regions could help bridge the gap between personalization and scalability. Indeed, molecular therapies able to target multiple patients may increase the attractiveness of investing in the development of ATMPs for rare genetic diseases. Proof-of-concept strategies developed for *LAMB3*-JEB, and for *COL7A1*-RDEB, underscore the potential of simultaneously targeting multiple mutations within a single EB-gene (Benati et al., 2018; Bonafont et al., 2021). Furthermore, a growing body of evidence across diverse genetic disease contexts supports the feasibility and economic sustainability of this approach (Rai et al., 2020; Pavel-Dinu et al., 2019).

Collectively, while gene- and cell-based therapies for EB, ichthyoses, and related genodermatoses have achieved remarkable milestones, their translation into widely accessible clinical solutions remains constrained by challenges related to stem cell targeting, efficiency, safety, durability and cost. Only through the integration of technological innovation with clinical and regulatory advances these debilitating disorders could fully benefit from the potential these advanced therapies.
